# An Error in the Use of Statistics

**Published:** 1902-09-20

**Authors:** 


### AN ERROR IN THE USE OF STATISTICS.

A common error in estimating the mortality of certain diseases is that which arises from striking a ratio between the number of deaths caused by the disease in question and the sum total of the deaths arising from all causes, instead of one between the deaths from the disease and the number of persons living, and although a moment's consideration is sufficient to demonstrate the nature of the error, it is one from which medical writings are by no means free. We are all familiar with the manner in which those who wish to show the frequency of small-pox before the days of vaccination turn to the old Bills of Mortality, and a modern instance, to which attention is drawn by a writer in the *Polyclinic*, is the attempt to show that cancer is not a senile disease. At the present day most people are agreed that as age advances the liability to cancer increases, but Mr. Roger Williams, in his treatise on Diseases of the Breast, takes the opposite view, and expresses himself somewhat forcibly on the subject. After citing various figures, he says: "These facts clearly show that cancer is not a senile disease, and that senility, *per se*, plays no essential part in its development. The contrary belief is a mere myth that by dint of continual repetition has gained widespread credence, without there being a particle of truth in it." Commenting on this, the *Polyclinic* says that it is certainly a matter to excite wonder that an observer so indefatigable as Mr. Williams should, dealing with precisely the same facts, have arrived at a conclusion so opposed to that of Dr. Tatham in the Registrar-General's report. Statistics are indeed untrustworthy if they can be made to support such contrary statements. The question, however, is, do they really do so, or is not Mr. Williams at error in the mode in which he uses them? In regard to this, the *Polyclinic* says:—

"Mr. Williams' method is to take the number of deaths occurring at different ages, and to count out the proportion caused by cancer. Thus he is able to show that during the two decades of 45 to 55, and 55 to 65, no fewer than one in every fourteen deaths was caused by cancer, whilst from 75 onwards only one in every forty-eight was so caused. This, however, is a wholly misleading way of stating the case. The question is, Are old people more prone to cancer? and the answer can only be given by estimating the proportion of deaths to the total number of those living at each age. When this is done, we find that during the decennium, 55 to 65, every thousand of those living supplied only 3·7 men and 4·5 women, whilst during the decennium 75 to 85 onwards the ratio was 6·7 men and 7·4 women. This is conclusive to the effect that the more advanced age is the one more liable to the disease. The asserted "myth" is not a myth at all, but a well-established fact. The pitfall into which Mr. Williams has fallen is forgetfulness of the fact that the proportion of deaths due to any one disease at any given age must always be in ratio with the liability of that period of life to other diseases. After the age of 65, other causes of death come into vigorous competition with cancer. A man's arteries may fail him, and he may die of apoplexy, or an attack of bronchitis which he would have thrown off in earlier life may easily prove fatal. After the age of 75, that unpreventable disease, old age, is upon him, and may probably not leave him the chance of developing cancer."

In Newsholme's book on "Vital Statistics" the following example of the fallacious character of this mode of reckoning mortality is given. Suppose a town of 100,000 with 2,000 annual deaths, of which 500 are caused by phthisis. Here the general death-rate is 20 per 1,000; the death-rate from phthisis is 5 per 1,000 living, and the deaths from phthisis form one-fourth of the total deaths. In another town having the same population the total deaths are 4,000, and therefore the death-rate 40 per 1,000 inhabitants; the deaths from phthisis are 1,000, and therefore the death-rate from phthisis is 10 per 1,000, but the proportion of the phthisical to the total mortality is one-fourth as before. In the second town, therefore, there is by the latter test apparently no worse condition, so far as phthisis is concerned, than in the first, although matters are really twice as bad.

